# A SYBR green I real-time polymerase chain reaction (PCR) assay for detection and quantification of *Trichomonas gallinae*

**DOI:** 10.1007/s00436-020-06887-x

**Published:** 2020-09-22

**Authors:** Zaida Rentería-Solís, Tran Nguyen-Ho-Bao, Shahinaz Taha, Arwid Daugschies

**Affiliations:** 1 Institute for Parasitology, Centre for Infectious Diseases, Faculty of Veterinary Medicine, University of Leipzig, An den Tierkliniken 35, 04103 Leipzig, Germany; 2 Albrecht-Daniel-Thaer Institute, An den Tierkliniken 29, 04103 Leipzig, Germany

**Keywords:** *Trichomonas gallinae*,, Real-time PCR,, SYBR green,, Trichomoniasis,, Flagellates

## Abstract

*Trichomonas gallinae* are parasitic flagellates of importance in wild and domestic birds. The parasite is worldwide distributed, and Columbine birds are its main host. Current research focuses mostly on epidemiological and phylogenetic studies. However, there is still a lack of knowledge regarding parasite-host interaction or therapy development. Real-time PCR is a useful tool for diagnostic and quantification of gene copies in a determined sample. By amplification of a 113-bp region of the 18S small subunit ribosomal RNA gene, a SYBR green-based real-time PCR assay was developed. A standard curve was prepared for quantification analysis. Assay efficiency, linearity, and dissociation analysis were successfully performed. Specificity, sensibility, and reproducibility analysis were tested. This assay could be a useful tool not only for diagnostic purposes but also for future in vivo and in vitro *T. gallinae* studies.

## Introduction

*Trichomonas gallinae* is a flagellate parasite with a wide number of wild and domestic avian hosts (Forrester and Foster 2008). It infects the upper respiratory and digestive tract. Birds can present mild to fatal disease, also called trichomonosis (Narcisi et al. 1991; Forrester and Foster 2008; Amin et al. 2014). Asymptomatic infections (trichomoniasis) are also common. Trichomonosis is characterised mainly by mild to severe lesions in the oral cavity, throat and crop. Other organs like the liver, lungs and air sacs can also be affected. Severity of the infection is related to strain virulence (Narcisi et al. 1991; Forrester and Foster 2008). Domestic and wild columbiformes are the main host of *T. gallinae* (Villanúa et al. 2006; Bunbury et al. 2007; Forrester and Foster 2008; Amin et al. 2014; Quillfeldt et al. 2018). Birds of prey, particularly Falconiformes are also a major host (Krone et al. 2005; Forrester and Foster 2008; Amin et al. 2014; Quillfeldt et al. 2018). Additionally, *T. gallinae* has also been reported in other birds such as Psittaciformes and very occasionally, domestic fowl (Amin et al. 2014). Trichomonosis outbreaks can lead to considerable decrease in avian populations (Lawson et al. 2011). Therefore, *T. gallinae* is considered a parasite of importance in veterinary and wildlife conservation (Bunbury et al. 2007; Forrester and Foster 2008).

Current *T. gallinae* research focuses on molecular epidemiology of *T. gallinae* in birds, particularly Columbiformes (Villanúa et al. 2006; Bunbury et al. 2007; Forrester and Foster 2008; Amin et al. 2014; Quillfeldt et al. 2018) and birds of prey (Krone et al. 2005; Quillfeldt et al. 2018). However, there is still a lack of knowledge regarding parasite-host interactions, as well as drug therapies (Munoz et al. 1998; Zimre-Grabensteiner et al. 2011).

Polymerase chain reaction assay (PCR) is a trustworthy tool that has been quite useful in *T. gallinae* molecular epidemiology studies (Forrester and Foster 2008; Lawson et al. 2011; Amin et al. 2014; Quillfeldt et al. 2018). The use of such molecular approaches could also be applied to other areas of *T. gallinae* research such as therapy and parasite-host interactions. Real-time PCR is an alternative to conventional PCR with a wider variety of applications. Real-time PCR (RT PCR) has been proved to be a useful tool in veterinary parasitology including diagnostics, quantitative analysis and normalisation of gene expression (Zarlenga and Higgins 2001). While conventional PCR has been widely used in *T. gallinae* epidemiological and genetic studies (Forrester and Foster 2008; Lawson et al. 2011; Zimre-Grabensteiner et al. 2011; Amin et al. 2014; Quillfeldt et al. 2018), RT PCR has not been exploited in *T. gallinae* research. The objective of this study was to develop a suitable RT PCR assay based on SYBR green I dye to detect and quantify *T. gallinae*.

## Material and methods

### *Trichomonas gallinae* culture and DNA purification

*T. gallinae* were obtained from ATCC (Virginia, USA) Strain 30002™ cultured and passaged in Diamond-LYI broth supplemented with 10% of heat-inactivated bovine serum following the conditions described by Garber et al. (1986). DNA extraction was performed using a DNeasy Blood and Tissue kit (Qiagen, Hilden, Germany) according to manufacturer’s instructions.

### Oligonucleotide primer design and selection

A partial sequence (GenBank accession number: MK172846) of the *T. gallinae* 18S small subunit ribosomal RNA gene (18S rRNA) was used as template for primer design. Regions of homology between other *T. gallinae* 18S rRNA partial sequences (EU215372, KM095107, KX58400, MK172847, MK172845, MK172844, MK172843, MK932770) were identified using the BLAST algorithm (Altschul et al. 1990). Oligonucleotides were designed using the Primer3 software (Untergasser et al. 2012). The primers selected for this study consisted on Trig7F 5’-GGTGGAGCCTGTGGCTTAAT-3’ and Trig7R 5’-CCATGCACCACCAAAAGCAA-3’ (Trig7F from 976 bp to 995 bp, Trig7R from 1088 bp to 1069 bp, accession no. MK172846) and were predicted to amplify a 113-bp fragment of the 18S rRNA gene. Finally, the predicted amplicon sequence was compared with the GenBank data base using BLAST (Altschul et al. 1990).

### Conventional PCR and Real-Time (RT) PCR assays

Conventional PCR assay was performed as follows: PCR reactions consisted of 2.5 μl of DreamTaq™ Green Buffer ×10 (Thermo Fisher Scientific, Dreieich, Germany), 0.2 μM from each deoxynucleoside triphosphate, 0.3 μM of each primer (Trig7F and Trig7R), 2 U of DreamTaq polymerase (Thermo Fisher, Dreieich, Germany), and 3 μl of *T. gallinae* genomic DNA (gDNA) as template. Finally, DNA/nuclease-free water was added up to a final volume of 25 μl. Temperature conditions consisted an initial denaturation time of 95 °C for 5 min, followed by 40 cycles of denaturation at 95 °C for 30 s, annealing at 55 °C for 30 s, and extension at 72 °C for 1 min, and a final extension period of 72 °C for 7 min. PCR products were detected and visualised by gel electrophoresis.

Real-time PCR assay consisted of 12.5 μl of Maxima SYBR Green/Rox qPCR Master Mix (×2) (Thermo Fisher, Dreieich, Germany), 0.3 μM of each primer (Trig7F and Trig7R), 3 μl of template and DNA/nuclease-free water up to a volume of 25 μl. Negative template control (NTC) consisted of DNA/nuclease-free water as template and was added to every assay in triplicates. All reactions were run in triplicates. DNA amplification was achieved by an initial denaturation time of 3 min to 95 °C, 40 cycles of 95 °C for 15 s and 55 °C for 1 min. A dissociation curve was performed after amplification by gradual rise in temperature from 65 to 95 °C with fluorescence signal measurement every 0.5 °C. With exception of the reproducibility assay (see 2.5.), all reactions were performed in a CFX Connect Real-Time PCR Detection System (Bio-Rad, Feldkirchen, Germany). Similarly, fluorescence normalisation and data analysis (except for reproducibility assay, see 2.5.) were done by thermal cycler software Bio-Rad CFX Maestro 1.1 (Bio-Rad, Feldkirchen, Germany).

### Standard curve

A conventional PCR was performed as mentioned in point 2.3. PCR products of the expected size were cloned into a pCR® 2.1 vector using the TA Cloning™ Kit (Invitrogen, Dreieich, Germany) following the manufacturer’s instructions. Plasmid (pCR18S) DNA was prepared using the GeneJet Plasmid Miniprep Kit (Invitrogen, Dreieich, Germany) according to the kit instructions. pCR18S Plasmid minipreps were commercially Sanger sequenced (Microsynth Seqlab, Göttingen, Germany) to confirm successful ligation of the desired sequence.

Plasmid DNA concentration (ng/μl) was calculated using a NanoPhotometer® NP80 (IMPLEN, Munich, Germany). A number of pCR18S plasmid DNA copies were generated based on the plasmid size (4013 bp: 3900 bp vector and 113 bp insert) assuming a molar mass of 650 g/mol per bp. Finally, a pCR18S plasmid 10-fold dilution series was used to generate a standard curve.

### Specificity, sensitivity and reproducibility assay

Based on the MIQE guidelines (Bustin et al. 2009), specificity, sensitivity and reproducibility assays were performed.

The specificity assay consisted on a conventional PCR. Genomic DNA of *T. gallinae*, *Histomonas meleagridis*, *Giardia duodenalis* and *Cryptosporidium parvum* were used as templates. In order to test for specificity within the family Trichomonadidae, a RT PCR was performed with gDNA from *T. gallinae* and *Tritrichomonas* (*Tr.*) *foetus* as templates.

For the sensitivity assay, the limit of detection (LOD) was estimated. LOD estimation was performed by assessing serial 10-log dilutions of the pCR18S plasmid (from 10^6^ to 10^−1^). Afterwards, the serial dilutions were used as templates in a RT PCR. LOD cut-off value was taken as the lowest concentration of analyte giving a positive result within a 95% confidence interval (CI) (Bustin et al. 2009).

The reproducibility assay was done by RT PCR assays with serial pCR18S plasmid 10-log dilutions (3 × 10^6^ to 3 × 10^1^) and gDNA from Budgerigar (*Melopsittacus undulatus*) crop naturally infected with *T. gallinae*. The RT PCR assay was repeated six times in total by different laboratory personnel, on different days, and in two different PCR systems: CFX Connect Real-Time PCR Detection System (Bio-Rad, Feldkirchen, Germany) and MX3000P cycler (Stratagene, La Jolla, USA). RT PCR assays were performed as described above except when working with the MX3000P cycler. For this system, the addition of 10 nM of ROX (Thermo Fisher, Dreieich, Germany) per reaction was necessary.

## Results

### Real-time PCR standardisation and performance

**Fig. 1:**
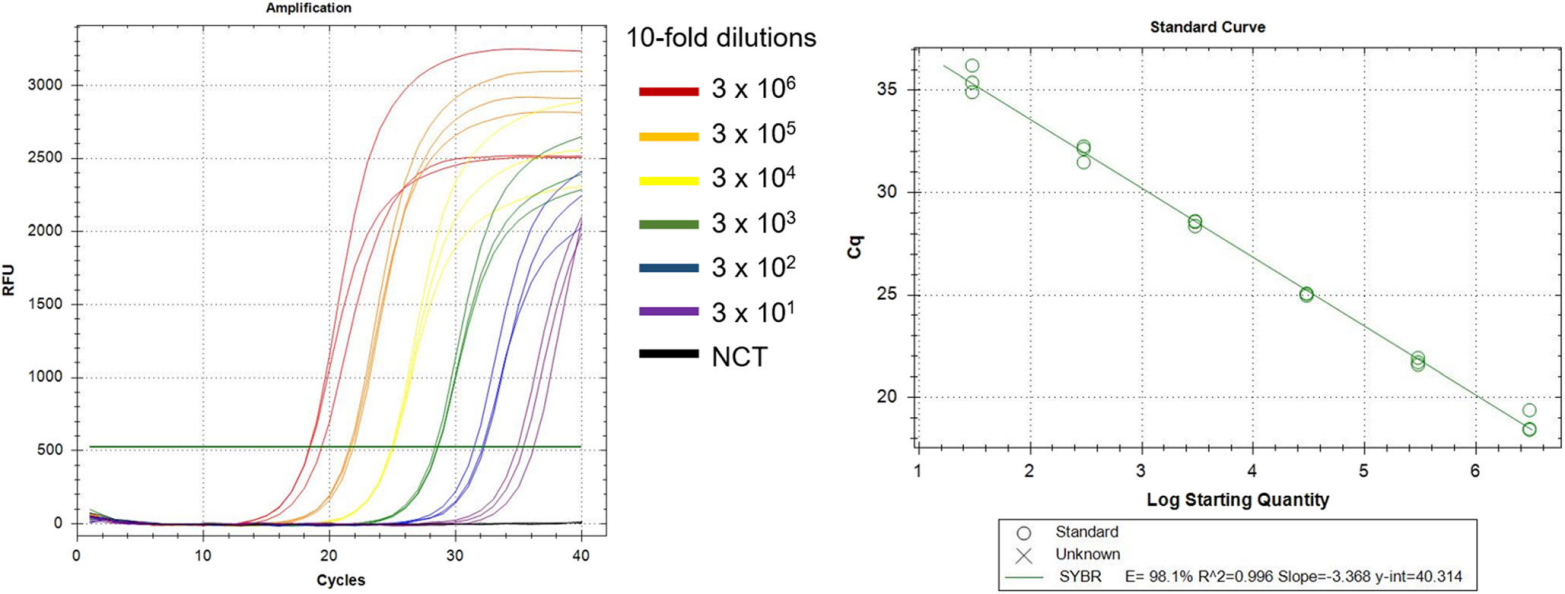
Amplification curve and standard curve analysis

Different concentrations of primers (100–900 nM) were tested for amplification reactions. Concentration of 300 nM of each primer per reaction was found to be optimal. Additionally, a series of PCR amplifications within an annealing temperature gradient estimated 55 °C as the most suitable value for this assay. Plasmid standard curves were constructed over a range of concentration copies from 3 × 10^6^ to 3 × 10^1^ (Fig. 1). Amplification efficiency values produced in this study were between the range of 90.7 and 101.3% (Fig. 1). These results are within the acceptable scope of 90 to 110% (Raymaekers et al. 2009; Maddocks and Jenkins 2017). Slope values of the standard curve calculated by linear regression analysis were from − 3.386 to − 3.573. The linearity of the assay was 3 × 10^6^ to 3 × 10^1^ with *R*^2^ values ranging between 0.993 and 0.999 (Fig. 1). Repeatability was assessed with intra-assay coefficient of variation percentages (CV%) below 1% (Table 1).

**Table 1:** Intra- and inter-assay coefficient of variance (%)

Template	Intra-assay*			Inter-assay		
	Ct**	SD	CV%	Ct**	SD	CV%
3 × 10^6	18.39	0.03	0.17	18.41	0.53	2.86
3 × 10^5	21.68	0.17	0.79	22.38	0.74	3.31
3 × 10^4	24.98	0.07	0.26	26.03	1.32	5.07
3 × 10^3	28.52	0.13	0.45	29.65	1.70	5.74
3 × 10^2	32.15	0.09	0.26	33.05	1.50	4.53
3 × 10^1	35.09	0.34	0.96	35.79	0.66	1.83
Crop sample***	26.06	0.14	0.55	25.73	0.47	1.83

*Results calculated for a single RT PCR run

**Mean of Ct values

***gDNA from crop tissue from a bird (*Melopsittacus undulatus*) naturally infected with *T. gallinae*

**Fig. 2:**
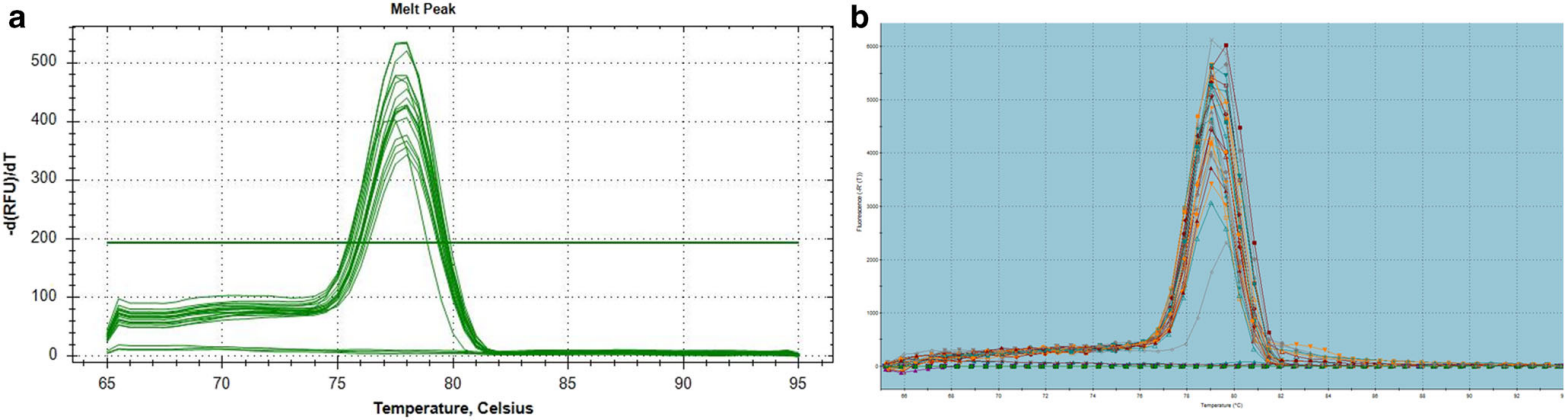
Dissociation analysis. A single melting peak produced during dissociation analysis in two RT PCR systems: (**a**) CFX Connect Real-Time PCR Detection System (Bio-Rad, Feldkirchen, Germany) and (**b**) MX3000P cycler (Stratagene, La Jolla, USA)

Absence of primer-dimer by-products was checked by dissociation curve analysis. Dissociation of the melting temperature for the 113 bp product occurred between 77.50 and 78 °C. Every RT PCR assay showed one clear single melt peak for each reaction (Fig. 2). No peaks or Ct values were observed in the NTC.

### Specificity, sensibility and reproducibility

Specificity of the assay parasites was checked against other protozoa after failure to visualise PCR products of the desired length (113 bp) for all samples except *T. gallinae*. However, *Tr. foetus* gDNA produced Ct values (23.49 ± 0.076) within the linear performance and CI was 95%. For sensitivity performance, LOD within 95% CI was determined to be around 3 × 10^2^ copies. In order to evaluate assay reproducibility, inter-assay CV% was estimated for every SC value (3 × 10^6^ to 3 × 10^1^) as well as one field isolate. Acceptable CV of less than 6% was produced for every template (Table 1).

## Discussion

We present in this study a real-time PCR assay for detection and quantitative analysis of *Trichomonas gallinae*. SYBR green I was selected as the reference dye for amplification tracking. SYBR green-based assays rely on dye binging to double strain DNA. This brings an extra challenge to the performance of the RT PCR since nonspecific products cannot be differentiated from real targets under these conditions (Zarlenga and Higgins 2001). An alternative to SYBR green dye is the addition of a fluorescence-labelled probe specific to the selected region that is directly related to the target amplification. Nevertheless, the addition of a probe is a costly step that makes of every RT PCR run more expensive than a SYBR green-based RT PCR. For this reason, a dye-labelled probe was not designed. However, future approaches with the latter option should be considered. A main challenge in primer design is the possible presence of by-products caused by primer dimer. The single melting peak during dissociation analysis showed no evidence of primer dimers in our assay. Additionally, efficiency values and linearity performance were satisfactory for all of the 10-fold dilutions (3 × 10^6^ to 3 × 10^1^) and RT PCR runs performed in this study which is in accordance to Maddocks and Jenkins (2017) and Raymaekers et al. (2009) and confirms suitability of the developed RT PCR.

A conserved region of the 18S rRNA gene was selected for amplification. The 18S ssu rRNA gene has been successfully used previously in *T. gallinae* epidemiology (Zu Ermgassen et al. 2016). Another region widely selected for *T. gallinae* phylogenetic studies is the ITS1-5.8S-ITS2 ribosomal region (Forrester and Foster 2008; Lawson et al. 2011; Zu Ermgassen et al. 2016; Quillfeldt et al. 2018) and it could be a suitable target for alternative RT PCR assays. Selectivity of the RT PCR assay was demonstrated by negative results for other flagellate parasites such as *H. meleagridis* and *G. duodenalis*, as well as the apicomplexan parasites *T. gondii* and *C. parvum*. However, a *Tr. foetus* sample was amplified successfully by the proposed assay. This indicates that our RT PCR protocol may not successfully discriminate amongst members of the family Trichomonadidae. However, *Tr. foetus*, although being relevant in bovine and feline species, is not found in the avian host and therefore discrimination appears not to be very relevant in terms of application. On the other hand, *T. vaginalis* and *T. tenax*, two species commonly associated with human infection, have been reported in birds (Quillfeldt et al. 2018). Unfortunately, for our study, no template from *T. vaginalis* or *T. tenax* was available. It is likely that the assay may not be able to discriminate these two *Trichomonas* species from *T. gallinae* either. Still, the percentage of *T. vaginalis* or *T. tenax* positive cases in birds is very low (Quillfeldt et al. 2018). It is not known if the presented RT PCR assay may also be suitable for quantification of other *Trichomonas* spp. which remains to be demonstrated by validation assays. Nevertheless, experimental studies with previously characterised *T. gallinae* strains would more likely be the main application for this newly developed RT PCR.

As previously mentioned, studies outside the epidemiological field are scarce for *T. gallinae*. Given the importance of *T. gallinae* in domestic and wild birds, the investigation of treatment options and parasite interaction with its hosts is pivotal. For this, in vivo and in vitro studies have been performed in the past (Munoz et al. 1998; Zimre-Grabensteiner et al. 2011; Amin et al. 2012). While in vivo models are still the best model to investigate biological and pathological process, in vitro assays represent an ethical alternative according to the 3R principle. Previous experimental studies relied on visual evaluation and quantification of lesions and parasite numbers in order to generate data (Narcisi et al. 1991; Munoz et al. 1998; Zimre-Grabensteiner et al. 2011; Amin et al. 2012, ). Such estimations are a good baseline; however, molecular approaches can add strength to the data analysis in future projects. For example, using RT PCR for quantification of *T. gallinae* gene copies can more accurately evaluate parasite susceptibility to a determined product or drug. There is an urgent need to study *T. gallinae* applying modern technology to avoid or reduce economic loss in the avian industry and to preserve endangered wild bird populations (Forrester and Foster 2008; Lawson et al. 2011). RT PCR can produce data to improve our current knowledge on *T. gallinae*, both by applied and basic research. Finding novel therapies, targeting anti-drug resistance, and understanding the parasite infection biology can bring insights into the disease and develop better preventive measures. The next step would be to extend the RT PCR applications beyond quantification of gene copies. For instance, RT PCR assays for analysis of gene expression have the potential to fill gaps in the understanding of an important and widely distributed parasite.

## References

[CR1] Altschul SF, Gish W, Miller W, Myers EW, Lipman DJ (1990). Basil local alignment search tool. J Mol Biol.

[CR2] Amin A, Bilic I, Berger E, Hess M (2012). *Trichomonas gallinae*, in comparison to *Tetratrichomonas gallinarum*, induces distinctive cytopathogenic effects in tissue cultures. Vet Parasitol.

[CR3] Amin A, Bilic A, Liebhart D, Hess M (2014). Trichomonads in birds – a review. Parasitology.

[CR4] Bunbury N, Jones CG, Greenwood AG, Bell DJ (2007). *Trichomonas gallinae* in Mauritian Columbids: implications for an endangered endemic. J Wildl Dis.

[CR5] Bustin SA, Benes V, Garson JA, Hellemans J, Huggett J, Kubista M, Mueller R, Nolan T, Pfaffl MW, Shipley GL, Vandesompele J, Wittwer CT (2009). The MIQE guidelines: minimum information for publication of quantitative real-time PCR experiments. Clin Chem.

[CR6] Forrester DJ, Foster GW, Atkinson CT, Thomas NJ, Hunter DN (2008). Trichomonosis. Wiley-Blackwell.

[CR7] Garber GE, Proctor EM, Bowie WR (1986). Immunogenic proteins of *Trichomonas vaginalis* as demonstrated by the immunoblot technique. Infect Immun.

[CR8] Krone O, Altenkamp R, Kenntner N (2005). Prevalence of *Trichomonas gallinae* in northern goshawks from the Berlin area of northeastern Germany. J Wildl Dis.

[CR9] Lawson B, Cunningham AA, Chantrey J, Hughes LA, John SK, Bunbury N, Bell DJ, Tyler KM (2011). A clonal strain of *Trichomonas gallinae* is the aetiologic agent of an emerging avian epidemic disease. Infect Genet Evol.

[CR10] Maddocks S, Jenkins R, Maddocks S, Jenkins R (2017). Chapter 4 – Quantitative PCR: Things to consider. Understanding PCR, A Practical Bench-Top Guide.

[CR11] Munoz E, Castella E, Gutierrez JF (1998). In vivo and in vitro sensitivity of *Trichomonas gallinae* to some nitroimidazole drugs. Vet Parasitol.

[CR12] Narcisi EM, Sevoian M, Honigberg BM (1991). Pathologic changes in pigeons infected with a Virulent *Trichomonas gallinae* strain (Eiberg). Avian Dis.

[CR13] Quillfeldt P, Schumm YR, Marek C, Mader V, Fischer D, Marx M (2018). Prevalence and genotyping of *Trichomonas* infections in wild birds in central Germany. PLoS One.

[CR14] Raymaekers M, Smets R, Maes B, Cartuyvels R (2009). Checklist for optimization and validation of real-time PCR assays. J Clin Lab Anal.

[CR15] Untergasser A, Cutcutache I, Koressaar T, Ye J, Faircloth BC, Remm M, Rozen SG (2012). Primer3—new capabilities and interfaces. Nucleic Acids Res.

[CR16] Villanúa D, Höfle U, Pérez-Rodríguez L, Gortázar C (2006). *Trichomonas gallinae* in wintering Common Wood Pigeons Columba palumbus in Spain. Ibis.

[CR17] Zarlenga DS, Higgins J (2001). PCR as a diagnostic and quantitative technique in veterinary parasitology. Vet Parasitol.

[CR18] Zimre-Grabensteiner E, Arshad N, Amin N, Hess M (2011). Genetically different clonal isolates of *Trichomonas gallinae*, obtained from the same bird, can vary in their drug susceptibility, an in vitro evidence. Parasitol Int.

[CR19] Zu Ermgassen EKHJ, Durrant C, John S, Gardiner R, Alrefaei AF, Cunningham AA, Lawson B (2016). Detection of the European epidemic strain of *Trichomonas gallinae* in finches, but not in other non-columbiformes, in the absence of macroscopic disease. Parasitology.

